# Health behaviour, health status and occupational prospects of apprentice nurses and kindergarten teachers in Germany: a cross-sectional study

**DOI:** 10.1186/s12995-016-0116-7

**Published:** 2016-05-21

**Authors:** Tanja Wirth, Agnessa Kozak, Grita Schedlbauer, Albert Nienhaus

**Affiliations:** Institution for Statutory Accident Insurance and Prevention in the Health and Welfare Services, Department for the Principle of Prevention and Rehabilitation, Pappelallee 33/35/37, 22089 Hamburg, Germany; University Medical Centre Hamburg-Eppendorf, Institute for Health Services Research in Dermatology and Nursing, Martinistraße 52, 20246 Hamburg, Germany

**Keywords:** Health behaviour, Health status, Job satisfaction, Mental disorder, Musculoskeletal disorders, Observational study, Student nurses

## Abstract

**Background:**

Apprentices in human service professions are exposed to emotional and physical stresses in their workplaces. Moreover, they are in the vulnerable phase of becoming an adult. Their lifestyle and health therefore seem to be particularly unstable. This study aims to evaluate and compare the health behaviour, health status and occupational prospects of apprentices in nursing and early childhood education and to identify factors associated with their physical and mental health.

**Methods:**

A cross-sectional study based on self-administered questionnaires was carried out at eight vocational schools in Hamburg, Germany. Four hundred two apprentice geriatric nurses, hospital nurses and kindergarten teachers/assistants participated (response rate: 99 %). Apprentices were compared in terms of their physical activity, dietary patterns, cigarette and alcohol consumption, body mass index, self-rated health, previous diseases, job satisfaction and occupational prospects. Factors associated with the participants’ musculoskeletal or mental disorders were identified using logistic regression.

**Results:**

Around 33 % of apprentice geriatric nurses and kindergarten teachers/assistants were overweight or obese. Fifty-five percent of geriatric nurses were smokers. Job satisfaction was lowest among hospital nurses. More than one third of the apprentices suffered from musculoskeletal or mental disorders. The ages of 23–26 years and mental disorder were associated with musculoskeletal disorders (OR 3.1, 95 % CI 1.4–6.7; OR 1.8, 95 % CI 1.1–3.1). Being an apprentice in early childhood education was associated with an increased chance of mental disorder (OR 2.9, 95 % CI 1.4–6.0). Additionally, musculoskeletal disorders, self-efficacy and irritation were associated with mental disorder.

**Conclusions:**

Differences between the occupational groups indicate the need for specific work-related health promotion for apprentices at an early stage in their careers. Future projects should focus on the implementation and evaluation of these measures.

## Background

Hospital and geriatric nurses as well as kindergarten teachers belong to the human service professions, which are becoming increasingly important in our society. Due to an ageing population in the course of demographic change, there is a growing demand for professional nursing care, which will be difficult to cover. Projections estimate that there will be a lack of up to half a million full-time employees in outpatient and inpatient care in Germany by the year 2030 [1]. Similarly, early childhood education has been expanded in Germany in recent years, also leading to a need for more employees in this occupational field. However, nursing professions in particular are not very attractive to graduates. One reason is that these professions are associated with high emotional and physical stress [2]. Besides increasing time pressure at work, dealing with suffering and dying patients is a major challenge in nursing. Kindergarten teachers are exposed to a continuously high level of noise and usually work in bent and twisted postures. Consequently, these professions show high rates of occupational disabilities and dropouts [3, 4], also among young employees [2]. Promoting health early in their careers could be one strategy to prevent high turnover in these professions.

Germany has a practically oriented apprenticeship system. In addition to school education, apprentices spend a large part of their vocational training in the workplace. Thus, apprentices in human service professions are exposed to both performance stress at school and a high degree of stress in their occupations. Moreover, most apprentices are still in the process of becoming an adult, a vulnerable phase in human life, during which one is susceptible to adjusting to an unhealthy lifestyle [5].

Results from the German Health Interview and Examination Survey (DEGS1) indicate that smoking and at-risk drinking are common among a representative sample of young adults aged 18 to 29 years. Forty percent of women in this youngest age group smoked; 36 % consumed alcohol at hazardous levels [6, 7]. According to the DEGS1, 30 % of young women in Germany are overweight [8].

Studies of students in general nursing support the concerns that they also do not follow a healthy lifestyle. A systematic review showed that the smoking rates among nursing students vary considerably depending on the country of study; from 3 % in Iran, to around 30 % in Great Britain, to over 60 % reported in one Australian survey [9]. With regard to Germany, studies consistently reported high smoking rates of over 40 % [10–15]. In a Scottish survey, 74 % of student nurses and midwives practiced harmful alcohol consumption [16]. Around one third of nursing students in Germany consumed alcohol at harmful levels [10]. The highest proportion (45 %) of overweight and obesity among nursing students was found in the US [17]. Rates reported by German studies of apprentices in the nursing field ranged from 20 to 32 % [12, 13]. A German survey reported that 58 % of apprentices at nursing schools consumed fast food at least once a week [14]. Symptoms of musculoskeletal disorders (MSD) and mental disorders such as depression or burnout have also been observed among nursing students [18, 19].

In Germany, separate vocational training is offered for the two disciplines of geriatric and hospital nursing. Most studies do not distinguish between these two professions; to the best of our knowledge, differentiated analyses among students with respect to health behaviour and health status are missing. Studies examining the health status and behaviour of young apprentices in early childhood education are also lacking. However, analyses of older employees in childcare showed that kindergarten teachers were less likely to be smokers compared to the general German population. In the same study, the prevalence rates for overweight and obesity were 41 and 18 % respectively [20]. Similarly, a study from Georgia reported that 50 % of female childcare providers were overweight or obese [21]. Most early childhood workers in a study from New Zealand believed that they have good nutrition and perceived their health as good or excellent. However, they reported an increase in physical symptoms, such as back pain, muscle strain and fatigue, since they started working in this setting [3].

Dual vocational training at schools and in workplaces provides a unique opportunity for the implementation of health promotion programmes that may increase the awareness of a healthy lifestyle and reduce the effects of occupational stresses. To identify needs for specific measures at vocational schools and workplaces, the health behaviour and status of apprentices must be determined. We have chosen the professions of geriatric nurses, hospital nurses and kindergarten teachers/assistants, as they work in settings with high emotional and physical demands; the constant and intense interactions with patients/clients require a high level of dedication and empathy. In addition, apprentices in these professions serve as role models for patients/clients with respect to their health behaviour. All three occupations also have in common the fact that they are mostly practiced by women and share the same required level of education (general certificate of secondary education).

The objectives of this study were (i) to describe the health status, health behaviour and prospects of apprentice geriatric nurses, hospital nurses and kindergarten teachers/assistants, (ii) to examine differences between these three groups and (iii) to identify factors associated with MSD and mental disorders of apprentices.

## Methods

### Study design, participants and procedures

A cross-sectional survey was carried out in Hamburg, Germany. Apprentice geriatric nurses, hospital nurses and kindergarten teachers/assistants were eligible for inclusion in the study. Apprentices in all years of vocational training, of both sexes and all ages were included at this stage. We calculated an estimate of the required sample size based on the prevalence of overweight, as defined by the body mass index (BMI). Overweight is an indicator for health behaviour and a factor influencing the health status. A mean prevalence of 30 % of overweight was assumed among apprentices [12, 13]. For a 95 % chance of our sample estimate being within five percentage points of this assumed prevalence (30 ± 5 %), a sample size of *n* = 322 was needed. We oversampled by 20 % in order to account for non-response and missing data. We therefore aimed to recruit approximately 403 apprentices.

Sixteen out of 20 vocational schools in Hamburg identified for the target professions were contacted at random. Eight of them gave their consent to participate in the study. The schools were responsible for selecting classes by convenience in which questionnaires could be distributed to apprentices during lessons. Apprentices were informed by a project member of the aim and content of the study, of measures to ensure confidentiality and of the voluntary nature of participation. By completing the questionnaires, participants provided their informed consent to take part in the study. Ethical approval was obtained from the Hamburg Medical Council Ethics Committee (# PV4649).

**Fig. 1:**
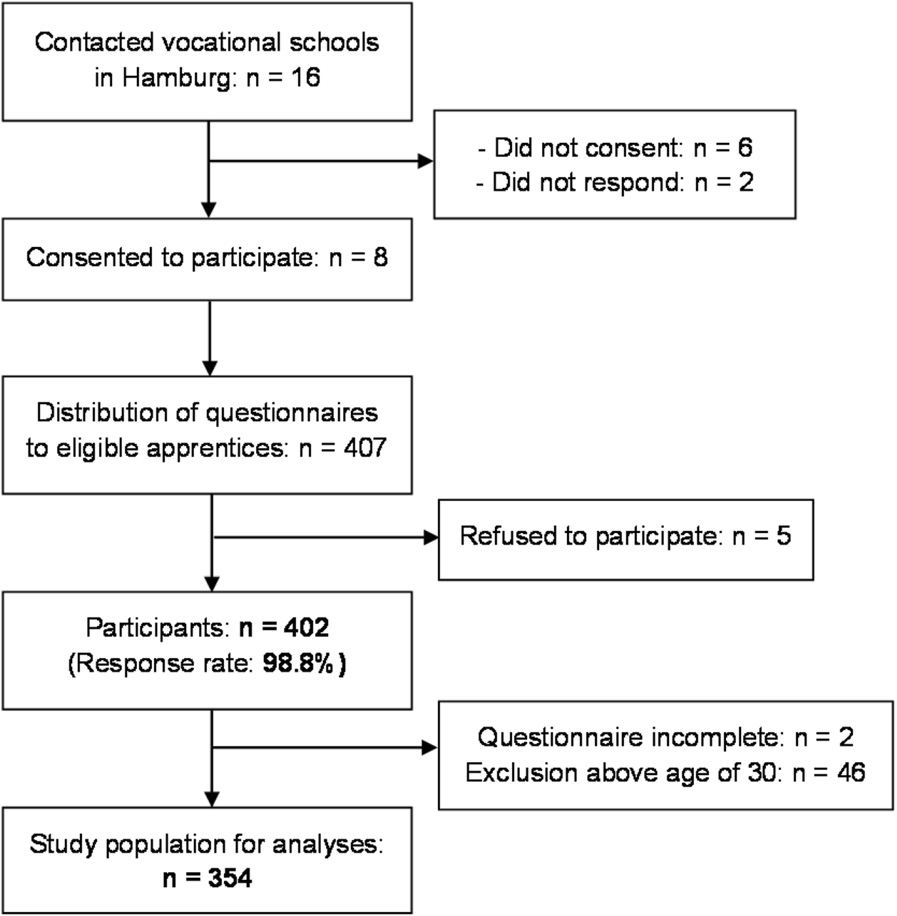
Flow diagram of the recruitment process

Data collection took place from January to March 2014. In total, 402 apprentices participated in the study (response rate: 98.8 %). The recruitment process is shown in Fig. 1. The age of participants varied greatly from 16 to 52 years. The health behaviour of younger apprentices differs considerably from that of older ones. For example, apprentices under the age of 30 years showed hazardous alcohol consumption (*χ*^2^ = 9.9 [df = 1], *p* <0.01) and unfavourable dietary patterns (*χ*^2^ = 11.4 [df = 2], *p* <0.01) significantly more often than their older counterparts. The study sample was therefore reduced to participants aged 16 to 30 years (*n* = 354). We set the cut-off at the age of 30 years to ascertain its comparability with the age group of 18 to 29-year-olds applied in the DEGS1 survey [6–8].

### Measures

A self-administered questionnaire was compiled from validated scales and single items which were applied in previous studies. An overview of the scales and items used in the analyses is given in Table 1. The internal consistencies of the self-efficacy, irritation and job satisfaction scale were good (Cronbach’s α = 0.81 to 0.88) and comparable to validation studies [22–24]. The applicability and comprehensibility of the instrument was verified in a pre-test with one class of apprentice geriatric nurses (*n* = 15). This class was not included in the final study sample.

**Table 1:** Variables of the study instrument used in the analyses

Variables	Name and source of the original scales and items	α	Items (n)
Socio-demographic factors^a^			18
Health behaviour			
Physical activity	Health Questionnaire age 18–64 years [29]	–	1
Dietary habits	Food Frequency Questionnaire [30]	–	15
Smoking	Health Questionnaire age 18–64 years [7]	–	2
Alcohol consumption	AUDIT-C [31, 32]	–	3
Health status			
General health status	Survey in vocational schools in Bielefeld [36]	–	1
Diseases and complaints	Work Ability Index – Questionnaire [33]	–	6
Self-efficacy	Self-efficacy scale [24]	0.85	10
Irritation	Irritation scale [22]	0.88	8
Occupational situation and prospects			
Job satisfaction	COPSOQ: German standard version [23, 34]	0.81	7
Job strain and prospects	Additional questions (e.g., taking into account the current work situation, do you want to stay in this profession for the next five years?)	–	3

^a^Included age, sex, height, weight, migration background, socioeconomic status and year of education

*α* = Cronbach’s alpha, *AUDIT-C* Alcohol Use Disorder Identification Test, *COPSOQ* Copenhagen Psychosocial Questionnaire

Information on the socio-demographic characteristics of participants included age, sex, height, weight, migration background, socioeconomic status (SES) and year of education. Apprentices were classified according to their BMI (kg/m^2^) as underweight (<18.5), normal weight (18.5 to 24.9), overweight (25 to 30) or obese (≥30). Migration background was assumed if apprentices themselves had immigrated to Germany and at least one of their parents was not born in Germany or was not of German nationality, or if both parents had immigrated or were not of German nationality [25]. The SES was determined from the highest educational level of apprentices and the current parental occupation. The following scores were administered to educational levels: 1 = comprehensive school qualification, 2 = general certificate of secondary education, 3 = technical college entrance qualification, 4 = university entrance qualification and 5 = technical college entrance qualification or university entrance qualification plus university degree. Parents’ occupations were coded according to the International Standard Classification of Occupations (ISCO-88) [26]. By using the International Socio-Economic Index of Occupational Status (ISEI), values from 16 to 90 were assigned to these codes [27]. Scoring categories were defined by quintiles. Scores for educational level were added to scores for the parental occupation with the higher ISEI value. The SES was classified as low (2 to 4), middle (5 to 7) or high (8 to 10) [28].

The health behaviour of apprentices was assessed by physical activity, dietary patterns, smoking habits and alcohol consumption. Regular physical activity was defined as a planned, structured and ongoing activity for increasing physical fitness. For the analysis, activity levels were classified as “no physical activity”, “up to 2 h/week” and “regularly, at least 2 h/week” [29]. A food frequency scale, ranging from never to daily, was applied to examine the consumption of fifteen food items. Each item had an individual scoring system from 0 = deviant to 2 = optimal. Scores were added together and classified according to the tertiles of the index [30]. A distinction was made between daily, occasional, former and never smokers. Apprentices smoking ≥20 cigarettes/day were classified as heavy smokers [7]. The Alcohol Use Disorder Identification Test (AUDIT-C) was performed to detect hazardous drinking behaviour. The cut-offs were >3 for women and >4 for men [31, 32].

The health status was rated on a five-point scale ranging from “excellent” to “poor”. Questions regarding diseases they had experienced during the previous twelve months were adapted from the Work Ability Index. Response categories were predefined as “no”, “yes, own opinion” or “yes, physician’s diagnosis” [33]. Two scales evaluated mental health. The General Self-Efficacy Scale identifies resources for coping with life’s challenges. It consists of ten items on a four-point Likert scale, with higher scores indicating better self-efficacy [24]. The Irritation scale measures emotional and cognitive psychological strain in occupational contexts. A higher score on a seven-point Likert scale reflects greater irritation [22].

Job satisfaction, motivation to stay in the profession, as well as occupational stresses and wishes were considered as prospects. Job satisfaction was measured by seven items on a four-point Likert scale. Response categories were transformed to 0 = very dissatisfied, 33 = dissatisfied, 66 = satisfied and 100 = very satisfied [23, 34]. Intentions to stay in the profession for the next five years were predefined as “yes”, “no” or “don’t know”. Two open questions asked about occupational stresses and wishes for the work situation. Out of the free texts, entries categories were formed, which were analysed quantitatively.

### Statistical analyses

Frequency distributions and percentages as well as means with corresponding standard deviations (SD) were calculated. Differences between the three professional groups were analysed using the chi-square test for categorical variables. Fisher’s exact test was performed if the expected cell frequencies were less than five. Mean differences were evaluated using one-way ANOVA and Scheffé post-hoc tests. If assumptions of normal distribution and homogeneity of variance were not met, non-parametric tests were used. Associations between health-related factors and binary outcome variables (MSD and mental disorder) were examined by logistic regression. Firstly, all variables with a significance value of *p* <0.05 from univariate analyses were included in a hierarchical model. Secondly, variables with *p*-value <0.25 were added [35]. If the model did not improve significantly through the second step, a final model with all the variables from the first step was calculated using the “enter” method. Odds ratios with corresponding 95 % confidence intervals and explained variances (Nagelkerke’s Pseudo R^2^) were calculated from logistic regression analyses. Statistical significance was set at *p* <0.05. All statistical analyses were performed using SPSS version 20.

## Results

The socio-demographic characteristics of the study participants are presented in Table 2. More than 75 % of apprentices were female. Geriatric nurses were slightly older (23.2 SD 3.3 years) than hospital nurses (21.9 SD 2.4 years) and kindergarten teachers/assistants (21.7 SD 3.5 years, *p* <0.01). Twenty-seven percent of apprentices were overweight or obese. Among geriatric nurses and kindergarten teachers/assistants, this applied to nearly one third. The SES differed significantly between the groups (*p* <0.001).

**Table 2:** Characteristics of the study population

	Geriatric nursing (*n* = 130)	General healthcare and nursing (*n* = 142)	Early childhood education (*n* = 82)	
Items	n (%)	n (%)	n (%)	*p*-value
Age groups (years)				<0.001
16–19	20 (15.4)	24 (16.9)	24 (29.3)	
20–22	38 (29.2)	62 (43.7)	29 (35.4)	
23–26	46 (35.4)	49 (34.5)	20 (24.4)	
27–30	26 (20.0)	7 (4.9)	9 (11.0)	
Sex				0.07
Female	98 (75.4)	122 (85.9)	63 (76.8)	
Male	32 (24.6)	20 (14.1)	19 (23.2)	
Body mass index				0.15
< 18.5	4 (3.1)	9 (6.3)	7 (8.5)	
18.5–24.9	78 (60.0)	95 (66.9)	45 (54.9)	
25–30	26 (20.0)	26 (18.3)	18 (22.0)	
≥ 30	14 (10.8)	5 (3.5)	6 (7.3)	
Migration background				0.13
No	90 (69.2)	110 (77.5)	57 (69.5)	
Yes	38 (29.2)	28 (19.7)	25 (30.5)	
Socioeconomic status^a^				<0.001
Low	64 (49.2)	25 (17.6)	37 (45.1)	
Middle	52 (40.0)	79 (55.6)	36 (43.9)	
High	4 (3.1)	30 (21.1)	3 (3.7)	
Year of education				<0.001
1st	32 (24.6)	59 (41.5)	29 (35.4)	
2nd	66 (50.8)	35 (24.6)	53 (64.6)	
3rd	32 (24.6)	48 (33.8)	/	

^a^Derived from educational level of apprentices and parental occupation

Missing values: Body mass index *n* = 21 (5.9 %); Migration background *n* = 6 (1.7 %); Socioeconomic status *n* = 24 (6.8 %)

More hospital nurses regularly took part in physical activities (37 %, ≥2 h/week) compared to kindergarten teachers/assistants (31 %) and geriatric nurses (25 %, *p* = 0.16). The dietary patterns of geriatric nurses and kindergarten teachers/assistants tended to be unfavourable (41 and 46 %) more often than those of hospital nurses (28 %, *p* <0.05). The proportion of daily and occasional smokers was highest among geriatric nurses (55 %, *p* <0.01). Twenty-one percent were classified as heavy smokers (≥20 cigarettes per day), whereas only 12 % of hospital nurses and 4 % of kindergarten teachers/assistants were classified as such. Hazardous drinking behaviour was equally common (*p* = 0.69) (Table 3).

**Table 3:** Group comparisons of health behaviour, health status and occupational prospects

	Geriatric nursing (*n* = 130)	General healthcare and nursing (*n* = 142)	Early childhood education (*n* = 82)		
Items	n (%)	n (%)	n (%)	Chi^2^ (df)	*p*-value
Health behaviour					
Physical activity				6.5 (4)	0.16
No physical activity	33 (25.4)	22 (15.5)	18 (22.0)		
< 2 h/week	62 (47.7)	66 (46.5)	39 (47.6)		
≥ 2 h/week	32 (24.6)	52 (36.6)	25 (30.5)		
Dietary patterns				9.9 (4)	<0.05
Unfavourable	53 (40.8)	39 (27.5)	38 (46.3)		
Normal	36 (27.7)	49 (34.5)	22 (26.8)		
Favourable	26 (20.0)	42 (29.6)	18 (22.0)		
Smoking habits				17.6 (6)	<0.01
Daily	58 (44.6)	43 (30.3)	29 (35.4)		
Occasionally	14 (10.8)	7 (4.9)	7 (8.5)		
Former	17 (13.1)	15 (10.6)	5 (6.1)		
Never	41 (31.5)	77 (54.2)	41 (50.0)		
Hazardous alcohol consumption				0.7 (2)	0.69
Yes	53 (40.8)	60 (42.3)	38 (46.3)		
No	72 (55.4)	80 (56.3)	41 (50.0)		
Health status					
Self-rated health status				9.2 (4)	0.06
Excellent/very good	52 (40.0)	55 (38.7)	25 (30.5)		
Good	65 (50.0)	74 (52.1)	40 (48.8)		
Not so good/poor	11 (8.5)	13 (9.2)	17 (20.7)		
Diseases and complaints					
Mental disorder	50 (38.5)	44 (31.0)	40 (48.8)	7.9 (2)	<0.05
MSD	45 (34.6)	52 (36.6)	26 (31.7)	0.5 (2)	0.79
Neurological or sensory disease	47 (36.2)	39 (27.5)	25 (30.5)	2.3 (2)	0.32
Respiratory disease	34 (26.2)	48 (33.8)	23 (28.0)	1.9 (2)	0.38
Skin disease	33 (25.4)	47 (33.1)	23 (28.0)	2.0 (2)	0.37
Injury due to an accident	37 (28.5)	28 (19.7)	25 (30.5)	4.0 (2)	0.13
	Mean (SD)	Mean (SD)	Mean (SD)	F (df1/df2)	*p*-value
Self-efficacy (10–40)	29.3 (4.5)	29.2 (4.2)	28.1 (4.9)	1.9 (2/338)	0.15
Irritation (8–56)	23.4 (11.1)	21.9 (9.9)	24.7 (10.2)	1.8 (2/343)	0.17
Occupational prospects					
Job satisfaction (0–100)	62.1 (15.9)	57.4 (15.2)	68.2 (16.7)	12.3 (2/350)	<0.001

Missing values: Physical activity *n* = 5 (1.4 %); Dietary patterns = 31 (8.8 %); Hazardous alcohol consumption *n* = 10 (2.8 %); Self-rated health status *n* = 2 (0.6 %)

*SD* standard deviation, *df* degrees of freedom, *MSD* musculoskeletal disorders

Around half of the apprentices in each occupational group rated their health as good. Between 31 and 49 % of apprentices reported a mental disorder (*p* <0.05), either in their own opinion or according to a physician’s diagnosis. MSD were the second most prevalent complaint, with proportions exceeding 30 % (Table 3). The body regions most frequently affected were the back, knees or knee joints. Kindergarten teachers/assistants had the lowest self-efficacy and highest irritation. Differences between the groups did not reach statistical significance (Table 3). Irritation was significantly higher among apprentices in their third (24.0 SD 11.6) and second years (24.2 SD 10.5) of education than for those in their first year (21.1 SD 9.4, *p* <0.05).

Kindergarten teachers/assistants were significantly more satisfied with their job than geriatric and hospital nurses (*p* <0.001) (Table 3). Moreover, job satisfaction was highest among apprentices in the first year (68.6 SD 13.8), compared to the second (60.6 SD 15.6) and third years of education (53.1 SD 16.9, *p* <0.001). With respect to occupational stresses, geriatric nurses most frequently reported time pressure and stress (39 %), physical and mental exertion (30 %) as well as a lack of staff (26 %). Besides these aspects, a high proportion of hospital nurses perceived the team situation and interaction with colleagues as burdensome (26 %). Kindergarten teachers/assistants listed physical and mental exertion (35 %) as well as working hours (26 %) most often. The majority of them intended to stay in their profession for the next five years (62 %). This also applied to nearly half of geriatric nurses, but only to 36 % of hospital nurses. Motivation to stay in the profession was significantly lower in the third year of education (35 %) than in the second (47 %) and first years (57 %, *p* < 0.001). More than half of apprentices in all occupational groups demanded higher wages and better public standing for their profession. There was a strong wish for more staff among geriatric and hospital nurses (44 and 53 %). Kindergarten teachers/assistants requested an improvement in working atmosphere and teamwork (25 %).

The variables age, SES, mental disorder, irritation and job satisfaction were significantly related to MSD in univariate analyses and therefore included in a logistic regression model. The ages of 23–26 years (OR 3.1, 95 % CI 1.4–6.7) and mental disorder (OR 1.8, 95 % CI 1.1–3.1) were associated with MSD; the model explained 18 % of the variance in the outcome variable (Table 4).

**Table 4:** Variables associated with musculoskeletal and mental disorders

Musculoskeletal disorders (MSD)			
Independent variables	MSD n (%)	Crude OR (95 % CI)	Adjusted OR^a^ (95 % CI)
Age (years)			
16–19	17 (25.0)	1	1
20–22	38 (29.7)	1.3 (0.7–2.5)	1.2 (0.6–2.7)
23–26	58 (50.4)	3.1 (1.6–5.9)**	3.1 (1.4–6.7)**
27–30	10 (23.8)	0.9 (0.4–2.3)	1.0 (0.4–2.8)
Socioeconomic status			
High	16 (43.2)	1	1
Middle	67 (40.1)	0.9 (0.4–1.8)	1.0 (0.5–2.2)
Low	31 (24.8)	0.4 (0.2–0.9)*	0.5 (0.2–1.2)
Mental disorder			
No	60 (27.6)	1	1
Yes	62 (46.3)	2.3 (1.4–3.5)***	1.8 (1.1–3.1)*
Irritation (8–56)	/	1.04 (1.02–1.06)**	1.02 (1.00–1.05)
Job satisfaction (0–100)	/	0.98 (0.97–0.99)**	0.99 (0.97–1.01)
Mental disorder			
Independent variables	Mental disorder n (%)	Crude OR (95 % CI)	Adjusted OR^a^ (95 % CI)
Apprenticeship trade			
General healthcare and nursing	44 (31.0)	1	1
Geriatric nursing	50 (38.5)	1.4 (0.8–2.3)	1.4 (0.8–2.7)
Early childhood education	40 (50.0)	2.2 (1.3–3.9)**	2.9 (1.4–6.0)**
Socioeconomic status			
High	9 (24.3)	1	1
Middle	73 (43.7)	2.4 (1.1–5.4)*	2.2 (0.9–5.4)
Low	43 (34.7)	1.7 (0.7–3.8)	1.5 (0.6–4.1)
MSD			
No	72 (31.4)	1	1
Yes	62 (50.8)	2.3 (1.4–3.5)***	2.1 (1.2–3.7)**
Self-efficacy (10–40)	/	0.89 (0.85–0.94)***	0.93 (0.87–0.99)*
Irritation (8–56)	/	1.07 (1.05–1.10)***	1.05 (1.02–1.08)**
Job satisfaction (0–100)	/	0.98 (0.97–0.99)**	0.98 (0.97–1.00)

Multivariate logistic regression analysis to determine variables associated with MSD was performed with *n* = 318 participants (Nagelkerke’s Pseudo R^2^ = 0.18). Multivariate logistic regression analysis to determine variables associated with mental disorder was performed with *n* = 310 participants (Nagelkerke’s Pseudo R^2^ = 0.25)

^a^Adjusted for the other variables in the model

*OR* odds ratio, *CI* confidence interval; *p <0.05, **p <0.01, ***p <0.001

A logistic regression model for the outcome of mental disorder was formed with the variables apprenticeship trade, SES, MSD, self-efficacy, irritation and job satisfaction. Being an apprentice in early childhood education was associated with an increased chance of mental disorder (OR 2.9, 95 % CI 1.4–6.0). Additionally, MSD, self-efficacy and irritation were significantly associated with mental disorder; the model explained 25 % of the variance in the outcome variable (Table 4).

## Discussion

This study was the first to provide a comparison of the health of apprentices in nursing professions and early childhood education. Results show that the health behaviour of geriatric nurses and kindergarten teachers/assistants is particularly worrying. Overall, more than one third of participants reported MSD or mental disorder.

In the present study, only 25 % of geriatric nurses were engaged in sports (≥2 h/week). This proportion was considerably lower than among young adults aged 18 to 29 years (37 %) in a recent German survey (DEGS1) [29]. We applied a food frequency index to evaluate the dietary patterns of apprentices, which extends beyond a simple enumeration of consumption frequencies. According to this index, around 46 % of kindergarten teachers/assistants had unfavourable dietary patterns, although teaching a healthy diet is part of their curriculum in Germany. The prevalence of overweight and obesity ranged from 22 % for hospital nurses to 31 % for geriatric nurses. These rates were similar to those of the corresponding age group in the general female population in Germany (30 %) [8]. However, apprentices engaged mainly in technical and commercial occupations showed a lower prevalence of overweight and obesity (19 %) [36].

We detected high smoking rates among geriatric nurses (55 %) and kindergarten teachers/assistants (44 %) which exceeded the smoking rate of 40 % found among young female adults in the general population [7]. However, our findings are in line with the results of studies of nursing students in Germany (smoking rates over 40 %) [10–12, 14, 15]. When compared to international studies, our smoking rates were considerably higher [9]. The proportions of apprentices classified as hazardous drinkers ranged from 41 to 46 % in our study. These were slightly higher than in women from the general population aged 18 to 29 years (36 %) [6].

A considerable proportion of apprentices in our study had suffered from MSD during the previous twelve months (32 to 37 %). Also, high prevalence rates (25 to 53 %) were detected by other investigations of nursing students and professionals in early childhood education [3, 14, 18]. We found that apprentices aged 23 to 26 years were more likely to suffer from MSD than younger apprentices (16 to 19 years). However, the ages of 27 to 30 years was not associated with an increased chance of MSD, meaning that we could not prove a consistent age effect. Other investigations confirm an age trend [37, 38]. It cannot be ruled out that our findings result from the narrow categorisation of age groups chosen for the analysis.

In the present analysis, low SES was associated with a low prevalence of MSD. Previous studies have found inconsistent results. A review reported that low SES was associated with a higher prevalence of musculoskeletal pain [39]. One reason for the inconsistencies could be differences in the methods used to measure the SES. Additionally, we observed a significant correlation between age and SES. Therefore, age could have influenced the association between SES and MSD in our study.

An overview of systematic reviews found strong evidence that poor job satisfaction is a risk factor for the development of low back pain in adults aged 18 years and older [40]. In our study, no association between job satisfaction and MSD was found. Job satisfaction has probably not yet had any substantial influence on the physical health of apprentices, as they have just entered the profession.

Mental disorders measured in our study included several symptoms such as depression, anxiety and chronic insomnia. With frequencies between 8 and 18 % of mental disorders confirmed by a physician’s diagnosis, we observed higher twelve-month prevalence rates than the survey of the general German population (4 % in adults aged 18 to 29 years). This prevalence included only diagnosed depressions and no other symptoms which could explain the difference in prevalence rates [41]. Nevertheless, our results of mental disorders could indicate that apprentices suffer from high occupational stress in their workplaces and performance stress due to their involvement in exams at vocational schools, since we measured significantly higher irritation among apprentices in the second than in the first year of education. However, we did not control for academic performance in our study.

Kindergarten teachers/assistants had a 2.9-fold chance of experiencing mental disorder compared to hospital nurses. As the apprenticeship of early childhood education is provided by vocational schools without any reimbursement, a high financial burden is placed on apprentices. This could negatively affect their mental health status. However, differences observed between occupational groups could also be related to the SES. Individuals with a high SES have a lower risk of suffering from mental illnesses [41]. Hospital nurses had a significantly higher SES than the other groups, which could explain their better mental health status. In general, the dependency between SES and apprenticeship trade might have influenced the relationship between SES and mental disorder. In contrast to the literature, no association could be proven for the latter by logistic regression.

MSD and mental disorder were significantly associated in our study, although the direction of causality is unclear. Lewinsohn et al. [42] showed that physical illness was a significant risk factor for depression in older adolescents. There is also strong evidence for the coexistence of physical and mental illnesses in the general population [43].

### Strengths and limitations

The use of heterogeneous instruments to measure health behaviour and status makes a comparison of literature difficult, and also limits the generalisability of the results of this study. Because of the cross-sectional design, no conclusions on causal relationships can be drawn. In addition, results are based on self-reports of participants. When validating the food frequency index, Winkler and Döring [30] pointed out that participants tend to overestimate intake of foods with a healthy image. Furthermore, frequencies of overweight in adolescents are underestimated by studies relying on self-report [44]. For that reason, unfavourable dietary patterns as well as overweight and obesity are likely to be even more prevalent than identified by this study. It was not feasible to draw a random sample of apprentices from the participating schools. The decisions of schools on which classes could take part in the study could not be influenced, as these depended on the upcoming practical periods and exams. Since a convenience sampling strategy had to be used, the study sample might not be representative. We calculated ORs to identify factors associated with MSD and mental disorders. However, these ORs should not be interpreted as estimates of relative risks, as the outcomes were frequent and because of the temporal ambiguity due to the cross-sectional design of our study (Table 4).

Limitations due to a non-response bias can be excluded because of the high response rate of 99 %. The number of missing values was under 5 % for nearly all variables. Extensive socio-demographic information on participants was collected in order to identify possible confounders.

## Conclusions

According to the results of the study, it seems to be necessary to develop didactically sound teaching units aimed at strengthening the apprentices’ health and avoiding risk factors. Among geriatric nurses, a high smoking rate indicates that more information on the consequences of smoking, individual support in stopping smoking and explicit anti-smoking policies at vocational schools are required [9]. Ergonomic working practices should be actively propagated during vocational training to prevent work-related MSD. Adequate financial support during the apprenticeship might help kindergarten teachers/assistants in reducing stress. Good concepts of health promotion at nursing schools in Germany could already be identified through an ideas competition. Examples are an in-house training in kinaesthetics and a regular running training, accompanied by lessons on anatomy, physiology and nutrition [45]. Such concepts still have to be developed for the field of early childhood education.

Due to the small sample size, the results of this study should be verified. By including a population-based comparison group, it could be possible to distinguish whether apprentices or young adults in general are at higher risk of the investigated disorders. Furthermore, it should be examined whether health status and health behaviour influence the choice of a particular apprenticeship or whether the particular working and learning conditions influence the health status and behaviour of the young adults. In addition to age, apprenticeship trade, self-efficacy and irritation, which were identified as the most important factors associated with MSD or mental disorder, future studies could evaluate muscle activity and endurance, academic performance, major life events and social support for their association with the health status in apprentices [18, 40, 42].

Leaving aside temporal ambiguity, future projects should concentrate on planning and implementing health promotion measures in the three occupational fields, taking into account the particular circumstances of an apprenticeship. Scientific evaluation can ensure the methodological and content-related quality of these measures.
